# Anonymous Slanderers and Working Men

**Published:** 1888-06-23

**Authors:** 


### The Hospital

A Weekly Institutional Journal of

**Science, Medicine, Nursing, and Philanthropy.**

*For Hospitals, Asylums, Medical Practitioners, Students, Nurses, Families, Parishes, Congregations, and the Charitable Public.*

Vol. IV. No. 91.] [June 23, 1888.

### Anonymous Slanderers and Working Men.

After the meeting recently held at the Mansion House in advocacy of the Hospital Sunday collections, the Lord Mayor received a letter purporting to come from "a working man," in which the writer stated that certain reports of extravagance and mismanagement in hospitals had recently been published in the evening papers read by himself and his companions, which would, he said, tend to prevent their contributing to the support of these institutions. He enclosed a cutting from a small weekly paper, in one paragraph of which the journalist said he could not endorse Mrs. Gladstone's plea for our hospitals (published in The Hospital of June 9th, and subsequently quoted in many journals), because the management of them was marked by great waste, and they were made a butchering-ground for doctors and students. In the second paragraph, the same writer advised his readers to bestow their alms on two hospitals, which he named, and which, as he praised them, he had probably more knowledge of than of the others in the metropolis.

"These statements, and others like them," said the working man—who seemed to write in all good faith —"have never been publicly contradicted." They have not, but why? Because the writers and publishers of them take good care not to bring their wild accusations before the eyes of those competent to speak with any authority on the subject of hospital management and finance. Their catchpenny statements are meant for the ignorant, and they are careful not to support them with any statistics from which an honest mind, however uninformed, might gather a juster estimate of the value of their words. A few figures would soon controvert their accusations, as may easily be seen by anyone who will calculate the average cost of maintenance for each hospital patient from the tables given in our issue of June 2nd. Most people know that in all the great metropolitan hospitals the physicians and surgeons give their services for nothing, and the resident doctors are proud to obtain posts to which no pecuniary emolument attaches. In many institutions the probationer nurses even pay for their opportunities of learning, and in all our hospitals the salaries of the workers is very small, when the labour and responsibility that falls upon them are taken into consideration. The allowance for washing and uniform for the nurses is kept as low as possible; in fact, it may generally be said that the expenses of hospital maintenance are as small as the requirements of efficiency allow.

We make this statement fearlessly. Very few hospital managers or secretaries have any reason to dread the fullest investigation. In only one case, so far as we remember, has the demand for an inquiry into the finances of a hospital been refused or evaded. We allude to the St. John's Hospital for Diseases of the Skin, against which very serious charges were brought in *Truth.* The editor of *Truth* is, however, a well-known man, and his method of procedure was thoroughly in accordance with the rules of journalistic warfare. He uttered no vague accusations, he gave no stab in the back. He stated his convictions plainly, brought them before the notice of the governors, and demanded that they should be disproved, if they could be disproved, in a court of law—a challenge which the hospital officials have finally decided to accept. This was all thoroughly legitimate and straightforward; if the paragraphist of the *Weekly Dispatch*, whose words so startled and grieved the working man who wrote to the Lord Mayor, had conducted his campaign on the same principles we should have said not a word against him. Honest accusation, for which the accuser will give his data, is one thing; vague anonymous abuse is another, and a very different one. These guerillas of the press, making parade of wisdom and knowledge before the eyes of those who cannot ascertain the truth or falsehood of their statements, are the most dangerous foes either a man or a system can have.

The press has a great, an almost terrible power, in our country. "A small drop of ink, falling upon a thought, produces that which makes thousands, perhaps millions, think." It must often surprise the writers themselves to learn how far their words reach, how often they are quoted, how religiously they are believed. But this makes it the more essential that no writer should make a statement or award praise or blame without full conviction of its justice—the conviction founded on assured knowledge of its accuracy. Of course there are statements made in our newspapers, little scraps of gossip about great personages, with regard to which it does not matter an iota whether they are true or false. If it is stated that the Queen, for instance, is learning Hindustani—or Sanscrit or Yahoo, for that matter—it will indeed interest her Majesty's loyal subjects, but neither Queen nor people will be greatly wronged by any inaccuracy in the statement. But if the policy of a cabinet or the value and honesty of a philanthropic cause be impugned, it is a very different matter. Then every word must be weighed, every fact verified, and the writer who, through either falsehood or carelessness tells untruths on such matters, should be drawn out of the shelter he gains from the anonymity of the newspaper press, and placed in any modern equivalent we can invent for the pillory which a century ago would have received him. We do not want our newspapers to be all flattery of either things or people. He who "prophesies smooth things" is usually uninteresting, and often worthless; but undeserved praise, however injurious, is less so than undeserved blame; the sycophant does less harm than the slanderer. The public demands, the public has a right to truth, nothing less and nothing more.

But this letter from a working man raises an issue apart from the newspaper cutting he encloses. He does not seem wholly convinced by the disparaging remarks he has read, but he is somewhat moved by them. This is simple enough. He says these statements have been made again and again, and reiteration gives a semblance of truth. "Say a foolish thing, but oft enough," remarks Mrs. Browning, "the same thing shall pass at last for absolutely wise, and not with fools exclusively." These slanders, assiduously poured into his ears, and "never publicly contradicted" in his hearing, have impressed him with a sad, vague distrust of the hospital system. The best way to combat this is to invite him to share in the management of hospitals through delegates chosen by himself and his fellow-workers. The plan of allowing working men who contribute to the hospitals to elect governors proportionate in number to the amount they subscribe has answered admirably in Sunderland and elsewhere; why should it not succeed in London? It gives them a personal knowledge of the working of hospitals, which invariably deepens their respect for and interest in them; and the accompanying privilege of sitting on the board of governors makes them more ready to give what they can spare towards the support of the institution. The workmen governors are no sleeping partners in hospital affairs. They accept their responsibility proudly, and, being representatives of the class which chiefly profits by the existence of hospitals, their suggestions as to the management of them are often shrewd and practical. On the other hand, they know how to plead the cause of hospitals to their comrades, and thus win contributions which would be refused to both peers and preachers. The working man will believe one of his own class where he would distrust anyone else, and the best way to convince him that hospitals are genuinely beneficent institutions is to show him a man whom he knows in the intimacy of daily life to be honest and sensible, taking part in their management and praising their work.

